# The effect of HTLV1 infection on inflammatory and oxidative parameters in the liver, kidney, and pancreases of BALB/c mice

**DOI:** 10.14814/phy2.15243

**Published:** 2022-04-04

**Authors:** Saeed Niazmand, Arezoo Gowhari Shabgah, Sara Hosseinian, Jamshid Gholizadeh Navashenaq, Ali Kamali, Mohammad Reza Khazdair, Yousef Baghcheghi, Mahdiyeh Hedayati‐Moghadam

**Affiliations:** ^1^ 37552 Department of Physiology Faculty of Medicine Mashhad University of Medical Sciences Mashhad Iran; ^2^ 394237 School of Medicine Bam University of Medical Sciences Bam Iran; ^3^ Noncommunicable Diseases Research Center Bam University of Medical Sciences Bam Iran; ^4^ 435898 Department of Infectious Diseases School of Medicine Jiroft University of Medical Sciences Jiroft Iran; ^5^ Cardiovascular Diseases Research Center Birjand University of Medical Science Birjand Iran; ^6^ Student Research Committee Jiroft University of Medical Sciences Jiroft Iran; ^7^ 435898 Department of Physiology School of Medicine Jiroft University of Medical Sciences Jiroft Iran

**Keywords:** HTLV1, inflammation, nitric oxide, oxidative stress, tissue

## Abstract

Viral infections are linked to the progression of inflammatory reactions and oxidative stress that play pivotal roles in systemic diseases. To confirm this phenomenon, in the present study, TNF‐α level and oxidative stress markers were examined in the liver, kidney, and pancreas of HTLV1‐infected male BALB/c mice. To this end, twenty BALB/c mice were divided into HTLV1‐infected mice that were inoculated with 1‐million HTLV1‐infected cells (MT‐2), and the control groups. Two months after inoculation, the peripheral blood, mesenteric lymph nodes, liver, kidney, and pancreas were collected after deep anesthetization of mice (ketamine, 30 mg/kg). The extracted DNA of mesenteric lymph nodes was obtained to quantify proviral load (PVL) using quantitative real‐time polymerase chain reaction (qRT‐PCR). The levels of lipid peroxidation, total thiol (SH), nitric oxide (NO), TNF‐α, catalase (CAT), and superoxide dismutase (SOD) activities were examined in the liver, kidney, and pancreases. Furthermore, histopathological changes in the liver and kidney were evaluated. In liver tissue, the levels of MDA, TNF‐α, and blood cell infiltration were significantly increased, and the levels of CAT and SOD were significantly decreased. In the kidney, a reduction in SOD, CAT, and total SH and an increase in MDA and NO were observed. In the pancreas, CAT activity, total SH, and SOD were decreased, and the levels of MDA and NO were enhanced. In terms of TNF‐α production, it has been shown that the level of this inflammatory cytokine was increased in the liver, kidney, and pancreas. The HTLV1 may have a role in inducing inflammatory reactions and oxidative stress pathways in the tissues.

## Graphical abstract

Viral infections are linked to the progress of inflammatory mechanisms and interruption of oxidative stress that play pivotal roles in systemic diseases. The HTLV‐1 may have a role in the induction of inflammatory reactions and oxidative stress pathways in the tissues.

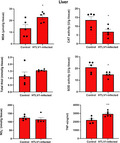

## 1 INTRODUCTION

Human T‐cell lymphotropic virus type 1 (HTLV1) is the first known human retrovirus belonging to the Delta Retroviruses and the subfamily of Orthoretroviridae (Mahieux & Gessain, 2009). Approximately 20 million people worldwide are infected with the HTLV1 virus (Proietti et al., 2005). The prevalence of HTLV1 is slightly higher in women than in men (1.02% and 0.66%) (Satake et al., 2012). The virus is endemic in certain parts of the world, such as southwestern Japan, Central Africa, the Caribbean, parts of South America, and Iran (Gessain & Cassar, 2012). According to epidemiological studies, 2%–3% of the population of Razavi Khorasan province in Iran is infected with HTLV1 (Shoeibi et al., 2013).

The virus causes an inflammatory‐neurological disease called HTLV‐associated myelopathy/tropical spastic paraparesis (HAM/TSP) and adult T‐cell leukemia/lymphoma (ATL). Infiltration of inflammatory cells and the presence of plaques, especially in the thoracic spinal, a spontaneous proliferation of T lymphocytes, increase in inflammatory cytokines such as IL‐2, IL‐4, IL‐6, IL‐15, TNF‐α, IL‐1β, and IFN‐γ has been observed in the serum and cerebrospinal fluid of HAM/TSP patients (Fuzii et al., 2014; Shoeibi et al., 2013). In addition, in some cases, infection with this virus is associated with disorders in various organs, including the lungs, cardiovascular system, urogenital system, and liver (Dias et al., 2018; Layegh et al., 2014; Lee & Kim, 2018; Santos et al., 2017). The HTLV1 virus activity in infected people is prolonged, and diseases associated with the virus are delayed and occur late in life. It has been reported that more than 90% of the virus carriers are asymptomatic (Goon et al., 2003a; Rafatpanah et al., 2007). In addition to activating inflammatory pathways, various studies show that infection with this virus is associated with increased oxidative stress and nitric oxide in plasma and various brain parts (Baydoun et al., 2015; Chaib‐Mezrag et al., 2014; Moghadam et al., 2018; Shomali et al., 2015). Oxidative stress results from an imbalance between oxidants and antioxidants in favor of oxidants. Increased formation of radical oxygen intermediates (ROIs) such as malondialdehyde (MDA) during oxidative stress leads to increased cell apoptosis, lipid peroxidation, protein denaturation, and DNA damage, which subsequently can play a substantial role in the development of various pathological conditions such as AIDS, inflammation, and cancer (Ivanov et al., 2017; Pohanka, 2013). Decreased antioxidant activity of glutathione (GSH), increased H_2_O_2_, and induced oxidative stress in the Jurkat cell line results from the HTLV1 Tax gene expression (Kinjo et al., 2010; Los et al., 1998). ROS induces inflammatory processes and the expression of chemokines such as IL‐6 and TNF‐α (Li et al., 2014). Chemokines can counteract NO activity, which is a vascular protective factor (De la Sierra & Larrousse, 2010). Different in vitro and in vivo studies have shown that inflammatory cytokines such as IFN‐γ, TNF‐α, and IL‐1 activate iNOS in macrophages and hepatocytes (Chanthaphavong et al., 2012). Considering the importance of the association of viral infections with the activation of the immune system, and the increase of oxidative stress in various tissues, and given that there is no study of the association of HTLV1 infection with oxidative stress, especially in peripheral tissues, in this study, oxidative and inflammatory changes in various organs, including the liver, kidney, and pancreas in HTLV1‐infected BALB/c mice, have been investigated.

## 2 METHODS AND MATERIALS

### 2.1 Cell line

The MT‐2 cell line, which is derived from the co‐culture of normal human lymphocytes with leukemia cells isolated from the ATL patient, was used in this study. For the cultivation of MT‐2 cells, RPMI 1640 medium (Caisson) and supplementations, including fetal bovine serum (FBS 10%), alanyl‐L‐glutamine (Gln 1%), and penicillin‐streptomycin mixture (pen/strep 0.1% Gibco) for the cultivation of MT‐2 cells were used to achieve an adequate number of cells.

### 2.2 Animal study design

This experimental study used female BALB/c mice weighing 20–30 g and aged 4–6 weeks. The animals were controlled in the laboratory and kept in the Center for Research and Care of Laboratory Animals with 12 h of light and darkness (from 7 to 19 h of light) and a temperature of 20–22°C and humidity 50%–55%. Standard dry food and drinking water were prepared and placed in the environment. After one week of adaptation, mice became inoculated with the virus by an intraperitoneal injection of PBS solution containing 10^6^ MT‐2 cells. All ethical principles of keeping and using animals that have been developed by the Institutional Ethics Committee were observed in the testing process. Mice were divided into two groups of 20 for MT2 cell injection: (1) As a control group, mice were given PBS buffer (2) and HTLV1‐infected group received 100 µl PBS solution containing 10^6^ MT‐2 cells. Two months after cell inoculation, mice were euthanized, then peripheral blood, mesenteric nodes, liver, kidneys, and pancreases were collected for further investigation. Mesenteric nodes were used for analyzing proviral load (PVL) by the TaqMan QRT‐PCR method. H & E staining was used for histological examination of the liver and kidney in terms of leukocyte infiltration. Liver, kidney, and pancreas tissue homogenates using phosphate buffer solution were prepared separately (10%, 0.1 g tissue in 1 ml PBS). Oxidative stress markers including MDA, total thiol groups, superoxide dismutase, catalase, and NO were measured in the liver, kidney, and pancreas tissues homogenates. TNF‐α were measured in the liver, kidney, and pancreases. The separated plasma of peripheral blood is used for the measurement of aspartate aminotransferase (AST), alanine transaminase (ALT), urea, creatinine, and uric acid by commercial kits (Pars Azmoon).

### 2.3 Assessment of HTLV1 infection

Total DNA from mesenteric nodes was isolated by a DNA extraction kit (Genet Bio). Subsequently, HTLV1 proviral DNA load (PVL) was evaluated to confirm the HTLV1 infection. Real‐time PCR was carried out using a PCR mixture (2 µl of extracted DNA, 1 µl of primers and probe, 2 µl of distilled water, 5 µl of TaqMan Master Mix) and a RotorGene Q 6000 machine (Corbett Research). The forward HTLV1 DNA primer, 5′‐CCCTACAATCCAACCAGCTCAG‐3, probe, 5′‐ FAM‐CTTTACTGACAAACCCGACCTACCC ATGGA‐ BHQ13, reverse HTLV1 DNA primer, 5′‐GTGGTGAAGCTGCCATCGGGTTTT‐3′, forward albumin primer, 5′‐CCTTGTCACTAGATGCAAAG‐3′, probe, 5′‐FAM‐CACATCACAACCACAACCTTCTCAG‐BHQ1‐3′ and reverse albumin primer 5′‐GACCATACGTGAAGACCTAA‐3′ were used. DNA concentrations of HTLV1 and the reference gene of albumin were quantified in accordance with standard curves. Finally, PVL per 10^4^ cells equaled ((copies number of HTLV1 DNA/copies number of albumin DNA/2) ×10^4^).

### 2.4 Biochemical assessment

#### 2.4.1 Assessment of lipid peroxidation through measuring malondialdehyde

To assess malondialdehyde (MDA; as a lipid peroxidation index), the TBARS (Thiobarbituric acid reactive substances) method was used. To begin, 0.5 ml of the homogeneous tissue mixture was transferred to a 10 ml centrifuge tube, followed by 3 ml of 1% phosphoric acid and 1 ml of 0.6% thiobarbituric acid (TBA) solution. The resulting mixture was heated in a boiling water bath for 45 min. After cooling the above mixture, 4 ml of n‐butanol was added, and after vortexing for 1 min, the butanol color phase was separated by centrifugation (at 20,000 rpm for 20 min), and the absorbance at 532 nm wavelength was read. MDA reacts with TBA to form a color complex with an absorption peak at a wavelength of about 532 nm.

#### 2.4.2 Measurement of total thiol groups (total SH)

DTNB (Ellman's reagent) was used to measure thiol groups. This reagent forms a yellow complex with SH groups (5‐Mercapto‐2‐nitro‐benzoic acid) with an absorbance peak of 412 nm. Then, 1 ml of Tris‐EDTA buffer (30 mM Tris, 3 mM EDTA, pH 8.2) was added to 50 µl of the homogeneous sample, and its absorbance was read at 412 nm subtracted from Tris‐EDTA buffer alone. Then 20 µl of DTNB reagent (in methanol) was added to it, and after about 15 min at room temperature, it was read to be subtracted to sample absorption.

#### 2.4.3 Measurement of superoxide dismutase activity

To assess superoxide dismutase (SOD) activity, tetrazolium and pyrogallol dye, 3‐(4,5‐dimethylthiazol‐2‐yl)‐2,5‐diphenyltetrazolium (MTT) were added to tissue homogenate. Tissue SOD destructs superoxide radicals that are the result of pyrogallol oxidation. In this method, the SOD amount was calculated by the reduction amount of tetrazolium dye, which is characterized by a peak absorbance of 535 nm.

#### 2.4.4 Measurement of catalase activity and nitric oxide

CAT is an enzyme that can degrade H_2_O_2_. To measure CAT activity, tissue homogenate and 1 µmol H_2_O_2_ were mixed. The absorbance of the prepared mixture was measured at 280 nm after 1 min from the initiation of the reaction. The determination of the NO level was accomplished by Griess reagent (Promega kit). The calibration curve was drawn following the measurement of absorption in the concentration range of 0.5 to 10 M of NaNO_2_ with an absorption peak of 550 nm. The collected tissues’ supernatants were mixed at room temperature with Griess reagent for 10 min with the substrate solution, followed by further incubation with the coloring solution for 10 min in the dark. The absorbance of the mixture was determined at 540 nm using a spectrophotometer.

### 2.5 Histopathological evaluation

After fixation of tissues in 10% formalin solution for 3 to 4 days, the tissues were dehydrated by passing through alcohols at different degrees and then molded. Sections with a 5 µm thickness were prepared on slides and then stained with the hematoxylin‐eosin (H & E) method. Slides were examined with an optical microscope, and images were provided. Changes were scored by a competent pathologist who was blinded to the groups according to the following: (A) no changes = 0, (B) mild changes = 1, (C) severe changes = 2.

### 2.6 Statistical analysis

Prism 8 software was used for data analysis. At first, data were analyzed to determine their normality of distribution by the Kolmogorov‐Smirnov test. Qualitative data were analyzed with the Pearson Chi‐Square test. The quantitative results were expressed as mean ± SEM, and to compare the mean difference between the tissues, one‐way ANOVA was used for parametric variables or Kruskal–Wallis for nonparametric variables. To compare data between both groups of the HTLV1 infected and control, the Mann–Whitney *U* test was used for nonparametric variables and the student *t*‐test for parametric variables. *p* < 0.05 was considered the level of statistical significance.

## 3 RESULTS

### 3.1 Effect of HTLV1 on provirus load (PVL)

The PVL results of this study confirmed infection of mice in the HTLV1‐infected group based on the calculated provirus load (PVL) in the mesenteric nodes of mice (Table 1).

**TABLE 1:** Proviral load (PVL) in mesenteric nodes of control and HTLV1‐infected groups

Groups	PVL = 10^4^ Mesenteric nodes cells
HTLV1‐infected	170.23 ± 60.2
Control	0

Note

PVL per 10^4^ cells of mesenteric nodes equaled (copies number of HTLV1 (DNA/copies number of albumin DNA/2) × 10^4^). The data are presented as mean ± SEM, *n* = 7.

### 3.2 Effect of HTLV1 on the levels of liver enzymes, urea, creatinine, and uric acid

Evaluation of liver enzymes in both groups has shown that the levels of ALT and AST in HTLV1‐infected groups compared with control groups were increased statistically (*p* < 0.05 and *p* < 0.01, respectively). The uric acid and creatinine levels in the serum of the HTLV1 infected group compared with the uninfected group were enhanced significantly (*p* < 0.01). Moreover, the urea concentration in HTLV1‐infected mice was also higher than control (*p* < 0.05) (Table 2).

**TABLE 2:** Comparison of level liver function enzymes (AST and ALT), urea, creatinine and uric acid in blood of control and HTLV1‐infected groups

Groups	ALT/GPT(U/L)	AST/GOT(U/L)	Urea (mg/dl)	U.A (mg/dl)	Cr (mg/dl)
Control	86.00 ± 4.15	1.26 ± 23.56	38.77 ± 5.02	5.27 ± 0.47	0.36 ± 0.02
HTLV1‐infected	156.67 ± 25.11^*^	2.73 ± 35.43^**^	52.67 ± 1.54^*^	8.70 ± 0.51^**^	0.45 ± 0.02^**^

Note

The data are expressed as mean ± SEM, *n* = 7. *p < 0.05 and **p < 0.01 compared to the control group.

### 3.3 Effect of HTLV1 on levels of lipid peroxidation, total SH, NO and TNF‐α, CAT and SOD activity in liver, kidney, and pancreas

**FIGURE 1:**
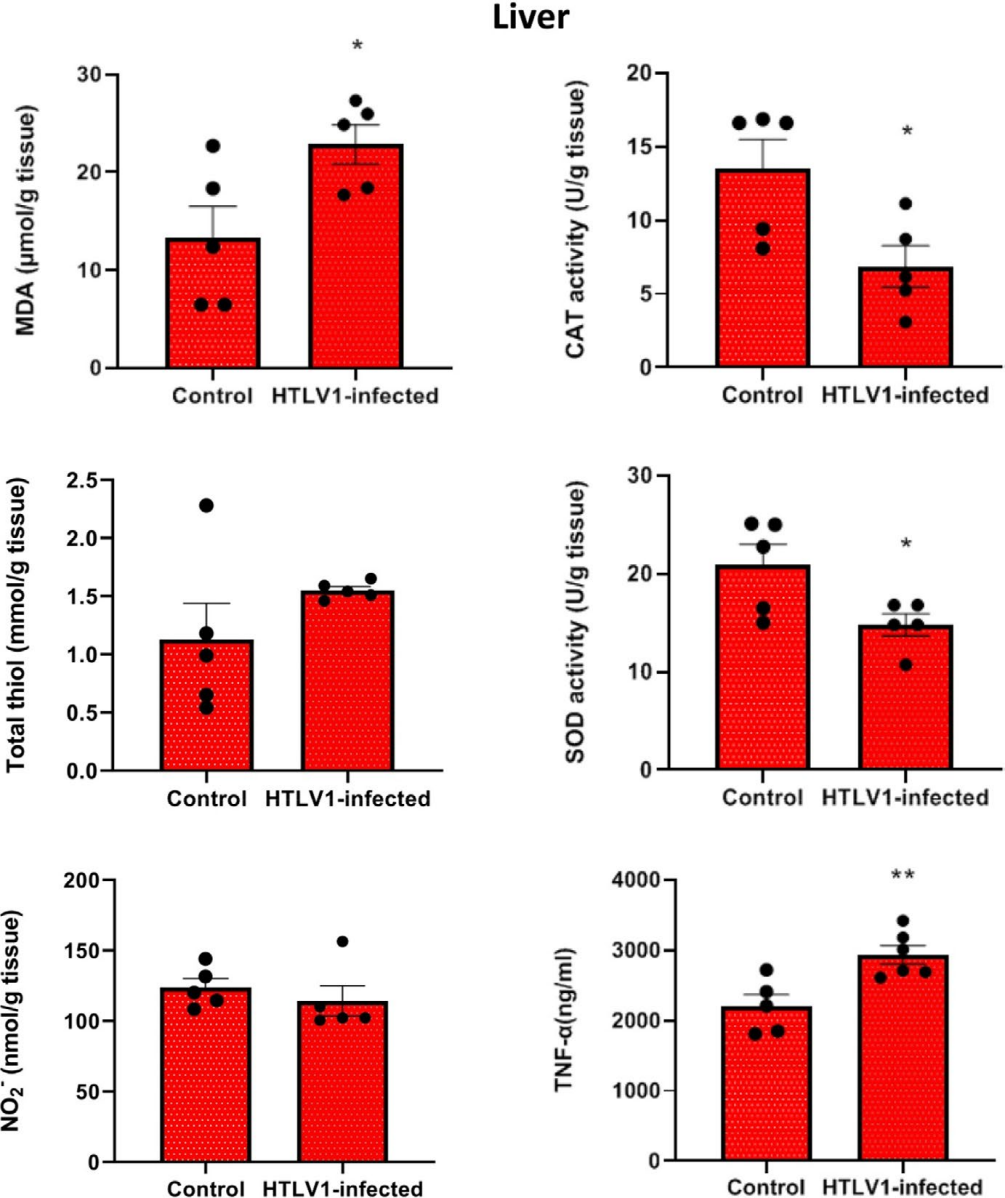
Comparison of malondialdehyde (MDA), catalase activity (CAT), total thiol (SH), superoxide dismutase (SOD), Nitric oxide (NO) and TNF‐α levels in liver tissue of HTLV1‐infected group compared with control group, using independent sample *t*‐test. Data are presented as mean ± SEM (*n* = 5 in each group). *p < 0.05 and **p < 0.01

The findings of this study revealed that the levels of MDA (*p* < 0.05) and TNF‐α (*p* < 0.01) in the liver of HTLV1‐infected group were significantly higher compared with the control group. The activity of CAT and SOD in liver tissue showed a significant decrease in the HTLV1‐infected group compared with the control group (*p* < 0.05). Total SH levels in liver tissue increased in the HTLV1‐infected group compared with the control group, but this increase was not significant (*p* > 0.05). NO levels in liver tissue decreased in the HTLV1 carrier group compared to the healthy control group, but this decrease was not significant statistically (Figure 1).

**FIGURE 2:**
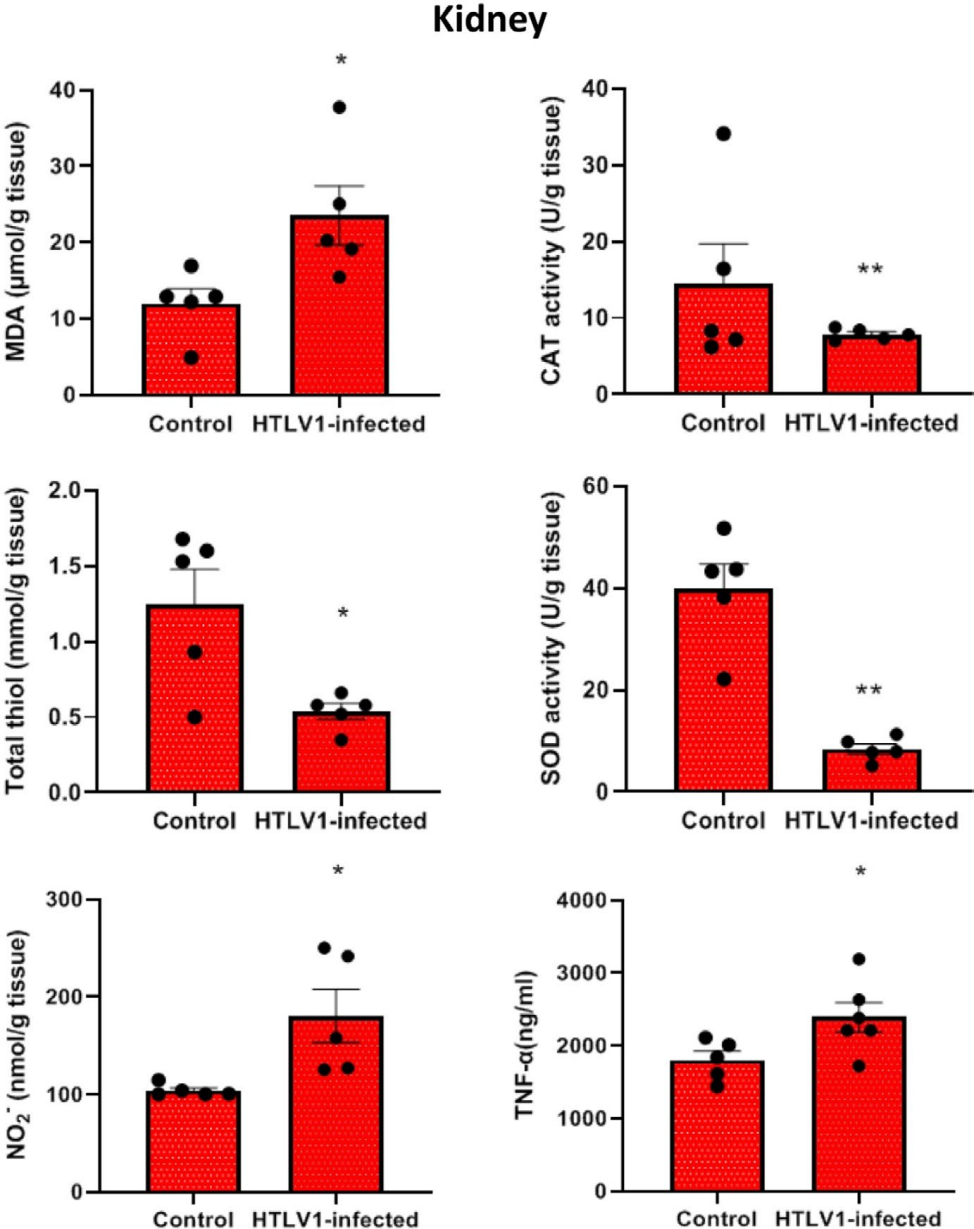
Comparison of malondialdehyde (MDA), catalase activity (CAT), total thiol (SH), superoxide dismutase (SOD), Nitric oxide (NO) and TNF‐α levels in kidney tissue of HTLV1‐infected group compared with control group, using independent sample *t*‐test. Data are presented as mean ± SEM (*n* = 5 in each group). *p < 0.05 and **p < 0.01

In the kidney, results showed that the levels of MDA, NO, and TNF**‐**α in the group of HTLV1‐infected were significantly increased compared with the control group (*p* < 0.05). The activity of SOD, CAT (*p* < 0.01), and total SH (*p* < 0.05) in kidney tissue in the HTLV1‐infected group showed a significant decrease compared to the healthy control group (*p* < 0.05). CAT activity in renal tissue decreased in the HTLV1‐infected group compared with the control group, but this decrease was not significant (Figure 2).

**FIGURE 3:**
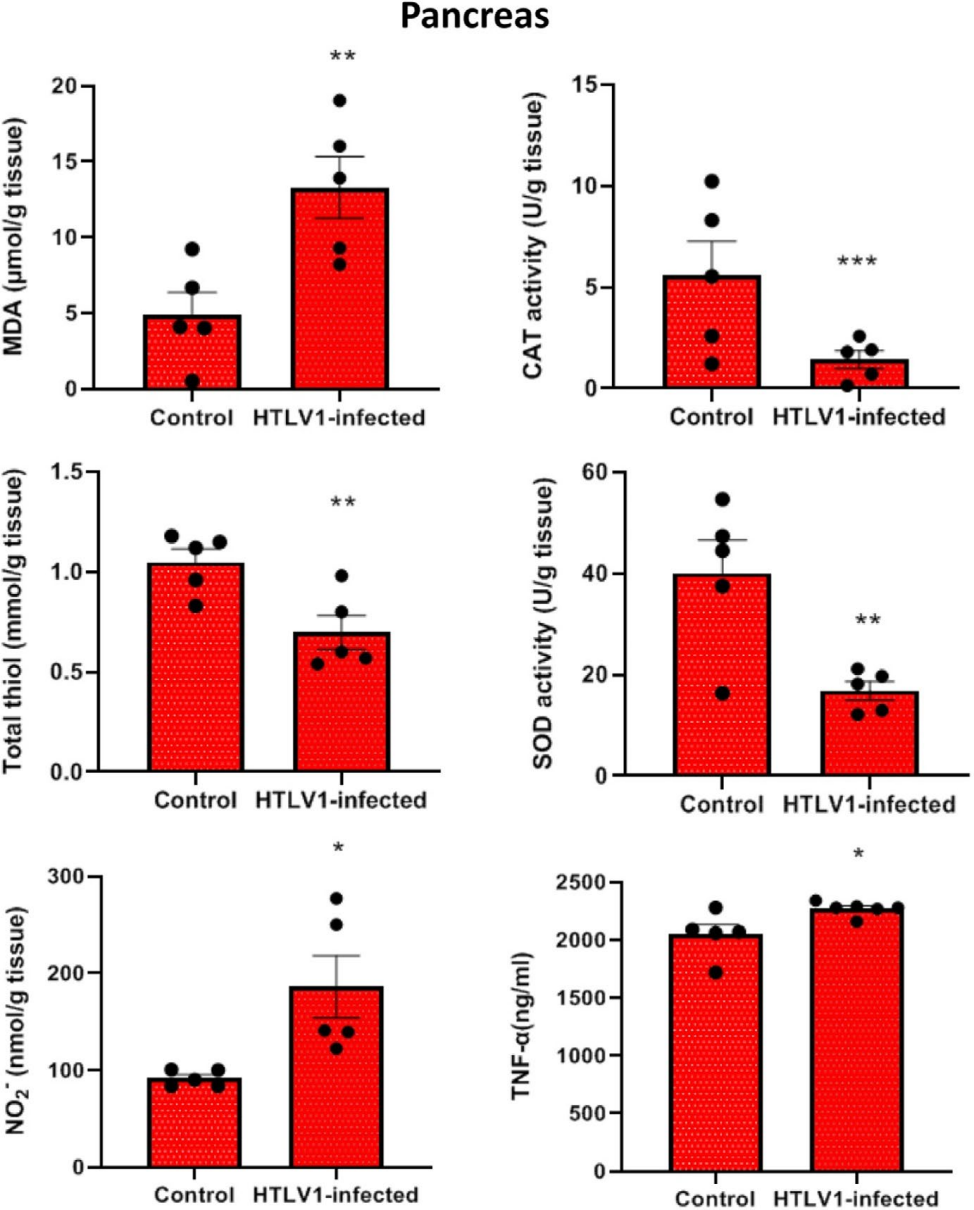
Comparison of malondialdehyde (MDA), catalase activity (CAT), total thiol (SH), superoxide dismutase (SOD), Nitric oxide (NO) and TNF‐α levels in pancreas of HTLV1‐infected group compared with control group, using independent sample *t*‐test. Data are presented as mean ± SEM (*n* = 5 in each group). *p < 0.05, **p < 0.01 and **p<0.001

In the pancreas, the levels of MDA (*p* < 0.01), NO (*p* < 0.05), and TNF ‐α in the group of HTLV1‐infected were significantly increased compared with the control group. The activity of SOD, total SH (*p* < 0.01), and CAT (*p* < 0.001) in pancreatic tissue showed a significant decrease in the HTLV1‐infected group compared with the healthy control group (Figure 3).

### 3.4 Effect of HTLV1 on liver and kidney histopathology

**FIGURE 4:**
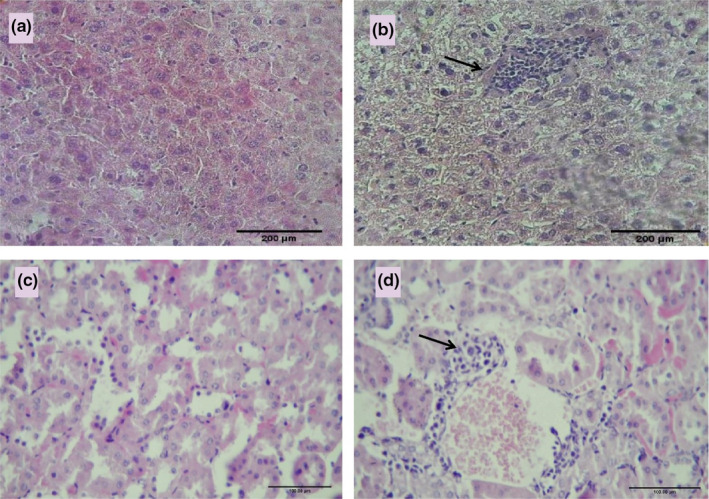
Photographs of a liver specimen in the control (a), HTLV1‐infected groups (b) and a kidney specimen in the control (c) and HTLV1‐infected (d) groups, Increased inflammatory infiltrate intra interstitial component of liver and kidney (in b and d) (magnification for each group; 40 × 10)

The histopathological examination of liver and kidney tissue after H & E staining showed that inflammatory factors and white blood cell infiltration in kidney and liver tissues increased significantly in the HTLV1‐infected group compared with the control group (*p* < 0.05) (Figure 4), (Table 3).

**TABLE 3:** Pathological finding scores in the control and HTLV1‐infected groups

Groups	Inflammation	
	Kidney	Liver
Control	0.33 ± 0.21	0.17 ± 0.17
HTLV1‐infected	1.3 ± 0.18*	0.71 ± 0.18*

Note

The data are expressed as mean ± SEM, *n* = 7.

* *p* < 0.05 compared to the control group.

## 4 DISCUSSION

This study detected provirus DNA by the TaqMan Real‐time PCR method in BALB/c mice mesenteric nodes two months after MT‐2 injection (IP). The mice peripheral blood mononuclear cells (PBMCs), mesentric nodes, and spleen tissues were used for infection confirmation in another our studies (Hedayati‐Moghadam et al., 2021; Moghadam et al., 2018).

The cycle threshold (CT) mean in the mesenteric nodes was higher than in the splenocytes and PBMCs. Fang and et al. detected HTLV1 DNA in C3H/HeJ mice's spleen, mesenteric nodes, and thymus 15 weeks after MT‐2 injection (Fang et al., 1998). In Tanaka study, 1 month after IP MT‐2 injection (106 or 107), the provirus was detected in most organs of the infected mice, including PBMCs, brain, salivary lymph nodes, lung, thymus, spleen, mesenteric lymph nodes, payer´s patches, liver, kidney, ovary, spinal cord, and submandibular glands. However, intravenously and orally injecting MT‐2 infected the liver and spinal cord, respectively (Tanaka et al., 2011). In Nagai's study, T lymphocytes PVL in HAM/TSP patients were reported 64 copies per 100 cells (Nagai et al., 2001).

The biochemical results of this study showed HTLV1 infection led to an increase in parameters of oxidative stress in the liver, kidney, and pancreas. If we want to go into details, in the liver tissue, the levels of MDA, TNF‐α, and blood cell infiltration were significantly increased, and the levels of CAT and SOD were significantly decreased. In the kidney, reductions in SOD, and total SH, as well as an increase in MDA and NO were observed. In pancreas, CAT activity, total SH, and SOD were decreased, and the levels of MDA and NO were enhanced. In terms of TNF‐α production, it has been demonstrated that this inflammatory cytokine is elevated in the liver, kidney, and pancreas.

Our previous study showed that the MDA level in the brain and lungs was increased (Hedayati Moghadam et al., 2018). In line with this finding, we have shown that the level of MDA in the liver, kidney, and pancreas was also elevated. Samadi et al. have indicated that oxidized dysfunctional HDL (HDLox) plays a pivotal role in immune activation and systemic inflammation in chronic viral infections such as chronic HTLV1 and HIV‐1. Therefore, it is concluded that HTLV1 infection by induction of systemic inflammation results in an altered level of lipid peroxidation (Samadi et al., 2019). Carvalho et al. (2015) have shown that infection with HTLV1 in women is associated with increased serum triglyceride and very‐low‐density lipoprotein (VLDL) levels. They also introduced viral infections, increased lipid profiles through receptor engagement, and applied them as a defensive mechanism. Koizumi et al. using pX transgenic mice showed that HTLV1‐infected rheumatoid arthritis (RA) patients are prone to cardiovascular disorders and atherosclerosis, resulting in hyperlipidemia (Babaie et al., 2018; Koizumi et al., 2005).

This study's result of plasma biochemical analysis demonstrated that high levels of ALT, AST, urea, and uric acid in HTLV1‐infected mice were increased compared with the control group. Using pX transgenic mice, Koizumi et al. showed that rheumatoid arthritis patients who are HTLV1‐infected are prone to atherosclerosis, and cardiovascular disorders resulting in hyperlipidemia (Babaie et al., 2018; Koizumi et al., 2005). Increasing plasma CPK in HTLV1‐infected mice may relate to the initiation of inflammatory reactions and the induction of neuromuscular disease or cardiovascular system injury.

Catalase (CAT) and superoxide dismutase (SOD) are enzymes that defend cells from free radical species attacks. Catalase hydrolyzes H2O2, and SOD is an oxidoreductase that serves to dismutate the superoxide anion (R Buettner, 2011). Our previous studies (Hedayati‐Moghadam et al., 2021; Moghadam et al., 2018) and this investigation have demonstrated that the activity of CAT and SOD in the mentioned tissue was reduced, which emphasizes the destructive role of HTLV1 infection on the free radical eradication system in vivo.

NO has been known as one of the most multipotent actors in the immune system. It is involved in the control and pathogenesis of tumors, infectious diseases, chronic degenerative diseases, and autoimmune disorders. Since NO can interact with multiple molecules such as nucleic acids, proteins, lipids, and carbohydrates, NO production affects immune responses (Bogdan, 2001). In this study, we have shown that NO production in the kidney and pancreas was decreased. This data shows that HTLV1 infection by induction of NO production affects the immune system and T cell response to expand viral infection.

CTL responses are crucial for virus clearance by the specific lysis of infected cells and the secretion of antiviral cytokines such as tumor necrosis factor‐alpha (TNF‐α). TNF‐α has been shown to be unregulated in HTLV1 infection (Goon et al., 2003b). These data are consistent with our findings that showed the level of TNF‐α was elevated in the liver, kidney, and pancreas. The high level of TNF‐α in HTLV1‐infected mice indicates that CNS pathogenesis might be a result of higher TNF‐α concentration (Goon et al., 2003b). Increasing plasma creatinine and TNF‐α in HTLV1‐infected mice may be associated with the initiation of inflammatory reactions and the induction of neuromuscular disease or cardiovascular system injury.

HTLV1 infection has encouraged oxidative stress and nitric oxide in the kidney, liver, and pancreas tissues and increased plasma levels of ALT, AST, urea, and uric acid in BALB/c mice. Furthermore, an increase in TNF‐α was observed in the kidney, liver, and pancreas of HTLV1‐infected male BALB/c mice. These data indicate that HTLV1 by induction of oxidative stress could affect the functions of organs and immune responses.

## CONFLICT OF INTEREST

The authors declare that they have no conflicts of interest.

## AUTHOR CONTRIBUTIONS

SN, AGS, and SH conceived and designed the experiments. SN, JGN, and AK performed the experiments, analyzed the samples, and drafted the initial version of the manuscript. MRK reviewed and edited the manuscript. MHM made substantial contributions to the study design and critically revised the manuscript. All authors read and approved the final version of the manuscript.
